# The Central Hospitals Board

**Published:** 1896-05-09

**Authors:** W. H. Allchin

**Affiliations:** Honorary Physician to the Westminster Hospital


### THE INSTITUTIONAL WORKSHOP.

### THE CENTRAL HOSPITALS BOARD.

By W. H. ALLCHIN, M.D., F.R.C.P., Honorary Physician to the Westminster Hospital.

THE Hon. Sydney Holland's address on a Central Hospital Board to the Hospitals Association on April 23rd deserved a better fate than it met with. Though I differ entirely from the main contention of the paper, I nevertheless recognise it as an earnest, capable, and, on the whole, the best proposal of its kind to meet what are universally admitted to be great difficulties. Once accept the principle of a Central Board, it is much better, as Mr. Holland points out, to adapt one of the various existing committees than to start a fresh one, which would be a long time in obtaining public confidence: and it is desirable that any such board should have the power of the purse. From such premises the conclusion to alter the Hospital Sunday Fund Committee so as to form such a board naturally follows. To those, however, who, like myself, object to the formation of a Central Board, it was a disappointment that the main principle was not discussed, and that the amendment of Mr. Ryan was allowed to obscure the issue. The result was partly due to Mr. Holland's too ready assumption that the principle of a Central Board is generally admitted, and that all that was required was to discuss how it should be carried out; but still more to the conduct of his friends, more particularly of the noble chairman, whose lengthy remarks were much lacking in lucidity, and who in the management of the meeting was singularly inept, giving the impression of wanting to rush the question, and being very uncertain what was and what was not the scope of the resolution and amendment. Mr. Acland's observation that opponents of the principle embodied in the resolution were "interested" in the maintenance of the present state of affairs was not redeemed, by saying that it was not meant "unkindly," from being a most improper remark, and one well calculated to rouse opposition and put the discussion on a low level. It may be worth while telling Mr. Acland that individuals whose lives and energies are spent in hospital work, whether they are paid or not, are not therefore less able to give an honest opinion on questions in which much technical knowledge is involved, than those well-intentioned amateurs who in the intervals of their own occupations are good enough to order other people's affairs.

It is a feature of the schemes proposed by the Lords' Committee, by the Charity Organisation Society, and by Mr. Holland, and dwelt on with much emphasis, that the Central Board should not possess any legal or compulsory powers, but that its efforts in the direction of improvement, or rather of giving effect to its wishes (not necessarily the same thing), should be by persuasion, advice, and comment in their annual report. The last-mentioned is nothing but a system of terrorism to be exercised by the press, which dearly loves a hospital scandal, and would jump at the chance of "going for" a hospital, whilst the imperfect information they could possess, based on the one side of the question put forth by the board, would be no bar to their avidity.

But in the absence of real power it is useless to talk of a Central Board "supervising" the hospitals of the metropolis. First of all, the work of "supervision" would be stupendous, and it is scarcely likely that the gentlemen who, for the love of it, week after week, with the greatest care and attention, manage the institutions with which they are connected in the best interest of those institutions, and with the fullest knowledge of their wants as well as of their ways and means, would willingly submit to a supervising board, no better, if half so well, informed as themselves. This is not to say that hospital management is perfect—far from it. It is often marked by dense stupidity and short-sightedness, but there is certainly no guarantee, and, to my mind, no probability, that these defects will be remedied by the collective wisdom of a central body, which must consist of similar persons to those who constitute the present house committees of hospitals, and on which the "faddist" is as certain to be represented. Without doubt the two greatest and most difficult questions connected with hospital administration, and which underlie to a great extent the financial difficulties, are, firstly, the out-patient department, and secondly, the establishment of fresh hospitals. Now in what way did the Lords' Committee deal with these matters? That Committee numbered among its members some of the ablest men, and was in a position to obtain the very best information from the most reliable sources tendered on oath.

No Central Board as hitherto proposed could put forward recommendations with half the force and weight possessed by the Lords' Committee, and yet they recommended that each hospital should deal with its out-patients in its own way, suggesting no guidance whatever; and definitely declined to advise that new hospitals should be made to show cause for their establishment to some authority. If that be an indication of what a really powerful body could do, what is to be expected from one which would have only this power of recommending? Its sense of responsibility would be lessened by the consciousness of its inherent weakness. What hospitals, that is, the large ones, want, is to be let alone to work out their own improvement. A great chance for the remedy of some faults, and the overcoming of some difficulties, was thrown away by the Lords' Committee, which wasted its energies in investigating petty personal squabbles, and eventuated in a futile report recommending the impracticable and mischievous scheme of a Central Board, a proposal which received no initiation or sanction from the accredited hospital witnesses who gave evidence before them, and no approval from the hospital representatives who were assembled to consider the suggestion after its promulgation. Are the hospitals now likely to go back on their policy?

